# CyberKnife® Radiosurgery as First-line Treatment for Catastrophic Epilepsy Caused by Hypothalamic Hamartoma

**DOI:** 10.7759/cureus.2968

**Published:** 2018-07-12

**Authors:** Pantaleo Romanelli

**Affiliations:** 1 Cyberknife Center, Centro diagnostico italiano, Milano, ITA

**Keywords:** epilepsy, hypothalamic hamartoma, seizures, gelastic, stereotactic radiosurgery, robotics, cyberknife, frameless, brain, pediatrics

## Abstract

Hypothalamic hamartomas (HH) are deep-seated lesions often associated with catastrophic epilepsy (an epileptic syndrome characterized by severe, drug-refractory seizures eventually leading to mental retardation and death). Radical microsurgical resection is not feasible for lesions located within the wall of the third ventricle inside the hypothalamus. Frame-based stereotactic radiosurgery has been reported as an effective treatment modality for small- to medium-size intrahypothalamic hamartomas, providing excellent seizure outcomes without lasting complications. This report describes the use of frameless image-guided robotic radiosurgery (CyberKnife® Radiosurgery System) as a first-line treatment in two children with catastrophic epilepsy induced by HH. Both patients experienced multiple-daily complex partial and gelastic seizures, as well as almost daily generalized seizures. The prescribed dose was 16 Gy (to the 65% isodose for case I; to the 70% isodose for case II). Lesional volume was 11.5 cc (case I) and 8.9 cc (case II). A steady reduction of the seizure frequency and severity was achieved after the treatment, starting about three months after the treatment. The generalized seizures disappeared within one year, while complete resolution of the gelastic seizures required up to 18 months. No seizure recurrence and no radiation-induced side effects or complications were witnessed over a follow-up period of ten years and eight months (case I) and nine years and seven months (case II) since the treatment. CyberKnife radiosurgery proved to be a safe and effective non-invasive first-line treatment in these two children with catastrophic epilepsy caused by HH.

## Introduction

Hypothalamic hamartomas (HH) are epileptogenic developmental malformations that grow inside the hypothalamus (sessile or intrahypothalamic) or mostly within the third ventricle (pedunculated or parahypothalamic). Their size is commonly less than 2 cm but larger or even giant lesions can be found as well. Severe, medically refractory seizures are common with HH that are broadly attached to the mammillary bodies, with thalamocortical spreading of the epileptic activity through the mammillothalamic tract [1-3]. HH neurons are characterized by intrinsic epileptogenic activity [4].

Early seizure onset in newborns and young children is often associated with catastrophic epilepsy, including drop attacks, gelastic and generalized seizures, and cognitive and behavioral deterioration leading to mental retardation [1,3]. This early onset form is aggressive and poorly responsive to medical therapy, requiring timely surgical intervention to prevent severe neuropsychological sequelae. Seizure onset later in life is typically associated with a milder course.

Surgical approaches include microsurgical resection through the transcallosal interforniceal, pterional or subfrontal translamina terminalis routes, microsurgical disconnection, endoscopic resection or disconnection, radiofrequency ablation, laser thermal ablation, and interstitial brachytherapy [5-8].

Stereotactic image-guided radiosurgery is an emerging non-invasive treatment option that provides a safe and effective treatment option [9]. This report describes the application of CyberKnife® radiosurgery (Accuray Incorporated, Sunnyvale, CA) as a first-line treatment in two children with catastrophic epilepsy induced by HH. CyberKnife radiosurgery system is an image-guided frameless device that delivers beams to the target in a non-isocentric fashion through a robotic linear accelerator (LINAC). CyberKnife radiosurgery provides the least invasive stereotactic radiosurgery modality available, with proven submillimetric accuracy despite the absence of a frame to immobilize the head [10].

## Case presentation

Two children (case I was eight years old and case II nine years old at the time of treatment) afflicted by catastrophic epilepsy induced by sessile HH not amenable to microsurgical resection were selected to undergo robotic frameless image-guided radiosurgery to improve seizure control. Both patients were affected since early childhood by multiple daily gelastic and generalized tonic-clonic seizures. Seizure control was poor despite several trials with multiple antiepileptic drugs (up to five medications combined) given at high doses. Both patients experienced prolonged post-ictal lethargy. Wechsler Intelligence Scale for Children (WISC), performed after full recovery from post-ictal state, indicated regular learning and memory development for case I and a moderate delay for case II. Case II was also affected by severe restlessness and poor school performance. Electroencephalography (EEG) was remarkable for severe abnormalities characterized by bilateral frontotemporal spikes. Magnetic resonance imaging (MRI) showed, in both cases, a medium-size non-contrast-enhancing hypothalamic lesion located in the wall of the third ventricle and growing into the interpeduncular fossa.

**Figure 1:**
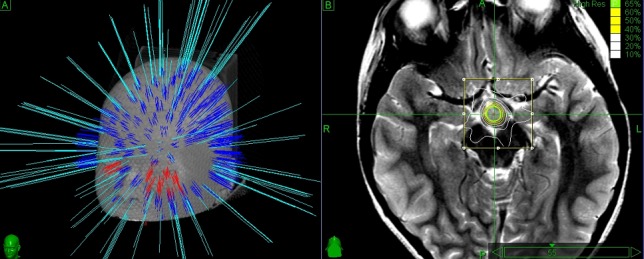
Case I. Left panel shows a 3D simulation of the beam pathways delivering 16 Gy prescribed to the 65% isodose. Frameless delivery allows the penetration of a wide number of beams below the orbito-meatal line. Right panel shows the isodose distributions on a T2W MR. An interpeduncular hyperintense lesion is visible just posterior to the optic tracts and attached to the mammillary bodies which are compressed, distorted and displaced posteriorly. Optic tracts, brainstem and hippocampi outside the 30% isodose, thus being spared by high-dose irradiation.

**Figure 2:**
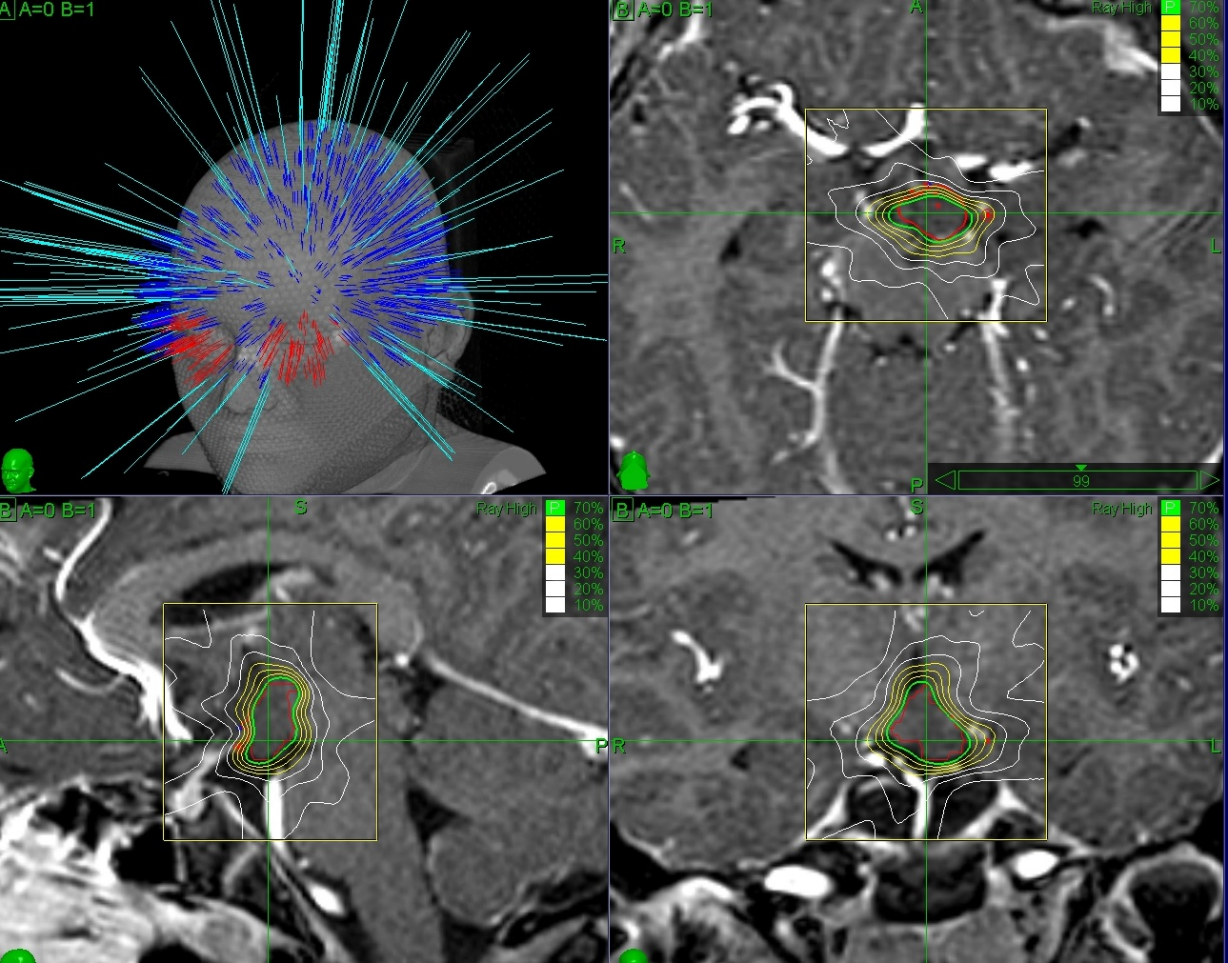
Case II. Treatment planning shown on T1W MR. The non-isocentric beam trajectories are visible on the top left. Isodose curves are visible in the axial, sagittal and coronal planes (top right, bottom left and bottom right, respectively).

CyberKnife stereotactic irradiation was delivered non-isocentrically to the HH as visible in T1- and T2-weighted volumetric MRI (Figures 1, 2). Thin-cut computed tomography (CT) was fused with the MR images and used for intraoperative localization. Digitally reconstructed scans obtained from the preoperative CT were fused with intraoperative digital X-ray scans providing the spatial reference frame needed for the accurate beam delivery by the robotic LINAC. Frameless single-session image-guided robotic radiosurgery was then performed. Due to the young patient age and frequent seizures, the treatment was performed in both cases under general anesthesia. Both patients received a single dose of 16 Gy, prescribed to the 65% (case I) and 70% (case II) isodose lines and delivered using, respectively, 175 and 151 non-isocentric beams. Treatment volumes were 1.10 cc (case I) and 0.89 cc (case II). Maximum doses were 24.43 Gy (case I) and 22.85 Gy (case II).

Nearby critical structures included the optic chiasm, pituitary gland, brainstem, mammillary bodies, mammillothalamic tract, and fornices. The dose delivered to the optic chiasm was kept below 5 Gy. In both cases, the mammillary bodies were found to be displaced, compressed, and distorted by the lesion (Figure 1). A single dose of dexamethasone (4 mg IM) was administered after the treatment.

The procedure was uneventful and well tolerated in both cases.

Case I experienced a brisk increase of gelastic and tonic seizures starting about two weeks after the procedure. Mild perilesional edema was found on MR imaging and scalp EEG showed a worsening of frontotemporal spike-and-wave discharges. After appropriate medication changes and a short course of dexamethasone, seizures and EEG activity returned to baseline. Starting three months after the procedure, a steady and progressive decline of seizure intensity and frequency was found. No more generalized seizures appeared after six months, while daily short gelastic seizures persisted up to 16 months after the procedure. A dramatic improvement of the quality of life and school performance was reported to follow the disappearance of generalized seizures and consequent post-ictal states. The child was found to be completely seizure-free (Engel grade Ia) 18 months after the procedure and has remained so for the following 10 years. Antiepileptic medications have been finally discontinued after two years of freedom from seizures (42 months after the procedure). Follow-up MRI performed six, 12, 18, and 24 months after radiosurgery failed to reveal changes in lesion volume and signal. No further signs of perilesional edema were found.

Post-radiosurgical course was uneventful for case II. Seizure frequency and severity began to decrease six months after the procedure and, after one year, the boy was found to be free from generalized seizures. He continued to experience occasional gelastic seizures for some time thereafter, then these too disappeared after 18 months. At this stage, a marked cognitive and behavioral improvement was noticed. Antiepileptic drug discontinuation was done after 12 months of complete seizure freedom (36 months after the procedure). The child has been seizure-free (Engel grade Ia) for nine years now. Follow-up MR showed a modest amount of perilesional edema appearing six months after the treatment and disappearing 18 months later. No changes in the lesion size were noticed.

In both cases, no visual, hypothalamic or pituitary disturbance was reported.

## Discussion

HH are associated with gelastic seizures, generalized tonic or tonic-clonic seizures, drop attacks, and partial seizures. Ictal and inter-ictal EEG are characterized by massive temporal and frontal lobe spikes but the true ictal onset lies deep within the hypothalamic lesion where GABAergic neurons with an intrinsic “pacemaker-like” behavior have been characterized [4].

The intrahypothalamic component typically lies in the wall of the third ventricle between the post-commissural fornix anteriorly, the mammillothalamic tract posteriorly, and the mammillary body inferiorly, which can be variably compressed and distorted together with the tuber cinereum, the preoptic and supraoptic nuclei, the optic chiasm, and the pituitary stalk.

Epileptogenic HH are invariably attached to one or both of the mammillary bodies. Spread of the epileptogenic activity and induction of generalized seizures require access to the thalamocortical loops via the mammillothalamic tract. The epileptogenesis of intrahypothalamic HH is therefore linked to the extensive connections with limbic neuronal networks, mediated by the mammillary bodies, which facilitate the spread of epileptic activity and the propagation of seizures.

The degree of seizure control is linked to the extent of the surgical intervention: incomplete resection, disconnection, or ablation of the HH can be associated with poor seizure control or seizure recurrence requiring further intervention. Early surgical attempts aiming to resect or disconnect supposed cortical foci failed to relieve the seizures while HH resection, disconnection, or ablation has been consistently associated with seizure relief [1]. The transcallosal-transforniceal approach provides excellent exposure of intraventricular hamartomas and remains today the favored microsurgical procedure [5].

Resective surgery is an excellent option for large pedunculated HH; the lesser the hypothalamic attachment, the lower is the chance to develop metabolic complication. Other approaches allowing partial or total resection or disconnection include the subfrontal translamina terminalis, the pterional transsylvian, and the subtemporal. Endoscopic resection and/or disconnection and radiofrequency or laser ablation have been used as well [5-8]. Surgical morbidity is overall limited, considering the challenging neural and vascular anatomy surrounding HH, but includes thalamocapsular infarcts with resulting hemiparesis or hemiplegia, oculomotor palsy, visual field deficits, short-term memory deterioration, hyperphagia, hyperthermia, hypothyroidism, syndrome of inappropriate antidiuretic hormone secretion (SIADH) and diabetes insipidus (DI) [5-8]. Complete seizure freedom is difficult to achieve but, in most cases, remarkable long-term improvements have been reported with near total abolition of disabling seizure types and enhanced cognitive and behavioral performances.

Stereotactic radiosurgery (SRS) provides an excellent approach to address intrahypothalamic lesions which are hard to resect totally without serious neuro-metabolic injury [9]. Most epileptogenic HH are small intrahypothalamic or medium-sized sessile intraventricular/inter-peduncular lesions. The two largest HH series report, respectively, a median size of 15 mm [7] and a mean size of 19 mm [2]. Radiosurgical treatment can be performed safely on HH with similar dimensions: these lesion volumes allow steep radiosurgical dose gradients providing relatively high doses to the HH while protecting the adjacent critical structures (optic chiasm, optic tracts, pituitary stalk, fornices, mammillary bodies, mammillothalamic bundle, hypothalamic nuclei, brainstem, hippocampi). No serious permanent complications, such as metabolic disorders, hemiparesis, cranial nerve deficits, or short-term memory deficits, have been reported after radiosurgery for HH.

The mechanism of action of SRS responsible for the seizure control is unknown. The lack of target necrosis as shown by follow-up MRs points toward a neuromodulatory effect induced either by gliosis, down-regulation of firing neurons and reduced vascular supply.

Temporary seizure worsening has been described as early as nine days after the procedure, in association with the delivery of prescribed doses greater than 16 Gy and is followed by progressive resolution [9]. A case of severe radiation-induced edema requiring long-term steroid administration despite a relatively low-dose (13 Gy prescribed to the 85% isodose line) has been also described. Regis et al. have recently published a prospective study on 57 patients with HH and drug-refractory epilepsy associated with severe cognitive and psychiatric comorbidities [9]. Follow-up longer than three years was available for 48 patients. Twenty-eight patients (58.3%) required a second treatment due to poor results after the first irradiation. Engel class I outcome rate was 39.6%, Engel class II was 29.2%, and Engel class III was 20% after three years. Overall, a 68.8% rate of complete or near complete seizure control (class I and class II) was achieved. Global psychiatric comorbidity was considered cured in 28%, improved in 56%, and stable in 8%, while further worsening was seen in the remaining 8%. No permanent neurologic side effects were reported (in particular, no memory deficit). Non-disabling transient poikilothermia was observed in three patients (6.2%).

Early treatment, as described in the current report, appears to be associated with favorable outcomes: both cases achieved seizure freedom (Engel class Ia) with remarkably improved social and cognitive performances. This is also the first report on the application of frameless robotic image-guided radiosurgery for HH. Frameless radiosurgery is a thoroughly non-invasive treatment offering submillimetric accuracy [10]. Furthermore, the absence of a stereotactic frame opens up a wide space for additional beam trajectories, thus enhancing the beam access to skull base or deep brain lesions. An additional degree of radioprotection is provided by the ability to hypofractionate the treatments if needed. The excellent seizure outcome here described was likely induced by the delivery of doses high enough to exert a marked antiepileptogenic effect and by the fact that treatment was not excessively delayed. It is rather common to see HH patients with catastrophic epilepsy leading to severe neuropsychological sequelae managed for long time with multiple (seemingly endless) medication changes, and being referred to epilepsy surgery centers only after years of devastating seizures. The two cases reported here illustrate how a thoroughly non-invasive treatment can greatly change seizure and life outcome, especially if applied early.

## Conclusions

CyberKnife radiosurgery provided a safe and effective treatment in two children with catastrophic epilepsy caused by HH. Early resort to stereotactic radiosurgery as a first-line treatment for epileptogenic HH not amenable to microsurgical resection should be discussed together with other options while counseling patients and families.
